# External quantum efficiency response of thin silicon solar cell based on plasmonic scattering of indium and silver nanoparticles

**DOI:** 10.1186/1556-276X-9-483

**Published:** 2014-09-11

**Authors:** Wen-Jeng Ho, Yi-Yu Lee, Shih-Ya Su

**Affiliations:** 1 Department of Electro-Optical Engineering, National Taipei University of Technology, No. 1, Sec. 3, Zhongxiao E. Rd, Taipei 10608, Taiwan

**Keywords:** Conversion efficiency, External quantum efficiency (EQE), Nanoparticle, Plasmonic scattering, Silicon solar cell

## Abstract

This study characterized the plasmonic scattering effects of indium nanoparticles (In NPs) on the front surface and silver nanoparticles (Ag NPs) on the rear surface of a thin silicon solar cell according to external quantum efficiency (EQE) and photovoltaic current–voltage. The EQE response indicates that, at wavelengths of 300 to 800 nm, the ratio of the number of photo-carriers collected to the number of incident photons shining on a thin Si solar cell was enhanced by the In NPs, and at wavelengths of 1,000 to 1,200 nm, by the Ag NPs. These results demonstrate the effectiveness of combining the broadband plasmonic scattering of two metals in enhancing the overall photovoltaic performance of a thin silicon solar cell. Short-circuit current was increased by 31.88% (from 2.98 to 3.93 mA) and conversion efficiency was increased by 32.72% (from 9.81% to 13.02%), compared to bare thin Si solar cells.

## Background

Photovoltaic energy is a viable renewable source of energy for coming generations. Unfortunately, the cost per unit of electricity generated by a photovoltaic system is higher than the retail price of electricity generated using conventional methods. Making power from photovoltaic devices competitive with other technologies, such as fossil fuels, will require considerable reductions in the cost of manufacturing. Current photovoltaic technology is based on bulk wafer-based crystalline silicon (C-Si) technology, which depends on the cost of Si materials and processing. Thus, the easiest way to reduce the costs of these devices is to reduce the amount of materials by producing thinner devices (thin Si solar cells; approximately 100 to 150 μm-thick) rather than traditional silicon solar cells (approximately 300-μm-thick). Many light-trapping methods have been proposed to achieve high efficiency without incurring high costs. Metallic nanoparticle plasmonic applications have been widely studied to enhance photovoltaic performance [1-4]. The resonance of most metallic nanoparticles is in the visible or infrared regions of the electromagnetic spectrum; however, this also depends on size, shape, and spacing of the metallic particles as well as the dielectric properties of the surrounding medium [5-7]. Most previous studies have shown that silver (Ag) and gold (Au) nanoparticles (NPs) can be used in bulk wafer-based C-Si solar cells or thin-film Si solar cells where the NPs are deposited on one surface of the solar cells [1,3,4,8-12]. However, few studies have examined the effects of metallic NPs deposited on the front and back surfaces of a thin Si solar cell [13].

This study fabricated solar cells with indium (In) NPs [14,15] on the front surface and Ag NPs on the rear surface. We then examined the degree to which photovoltaic performance was enhanced by the plasmonic scattering of In NPs and Ag according to external quantum efficiency (EQE) and measurements of photovoltaic current–voltage (I-V). EQE was enhanced at wavelengths of 300 to 800 nm thanks to In NPs and at 1,000 to 1,200 nm thanks to the Ag NPs. Short-circuit current was increased by 31.88% (from 2.98 to 3.93 mA), and conversion efficiency was increased by 32.72% (from 9.81% to 13.02%), compared to those of bare thin Si solar cells.

## Methods

A 250-μm-thick p-type (boron doped) Si wafer with resistivity of 1 to 10 Ωcm and (100) orientation was cut into small samples (1 × 1 cm^2^) and polished on one side for the fabrication of solar cells. The back side of the Si samples was then ground down to obtain Si samples of 120-μm-thick. After standard RCA cleaning, the thin Si samples were coated with a phosphorus liquid source (Phosphorosilicafilm; Emulsitone Co., Washington, NJ, USA) on the front surface using a spin-on film (SOF) technique at a speed of 6,000 rpm for 20 s. This was followed by prebake processing on a hot plate at 200°C for 5 min for the removal of solvents and 400°C for 10 min to promote cross-linking. Both sides of the samples were then capped with a 250-nm-thick SiO_2_ layer using e-beam evaporation and heated in a rapid thermal annealing (RTA) chamber under an N_2_ atmosphere at 900°C for 2 min in order to diffuse the phosphorus resulting in an n^+^-Si emitter approximately 0.4 μm in thickness. Following phosphorus diffusion, the samples were soaked in an HF solution to remove the SiO_2_ caps as well as the layer of phosphorus oxide. The diffusion profile was examined using secondary ion mass spectrometry (SIMS). We then deposited 20-nm-Ti/200-nm-Al films on the front surface using patterns of photo-resist. Finally, the samples were isolation etched in KOH solution using a photolithography process to obtain individual areas 4 × 4 mm^2^.

**Figure 1:**
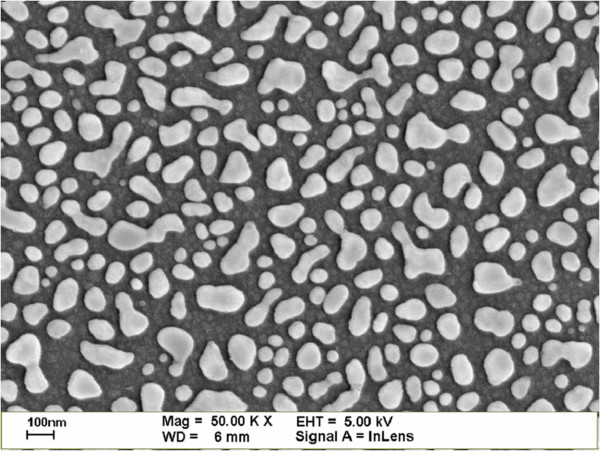
SEM image showing the sizes and profiles of Ag NPs.

**Figure 2:**
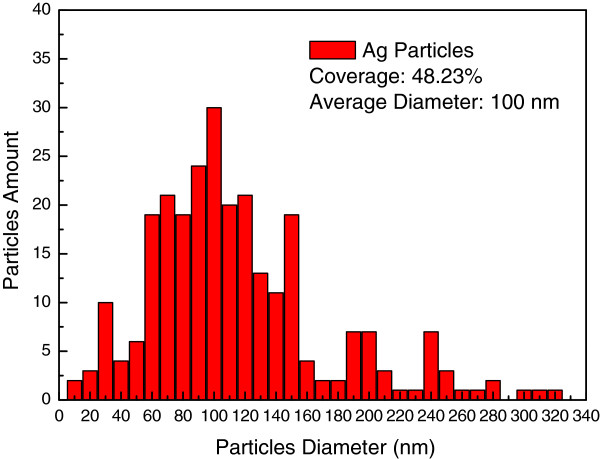
Ag NPs particle size distribution calculated by analyzing the SEM image using J-image software.

**Figure 3:**
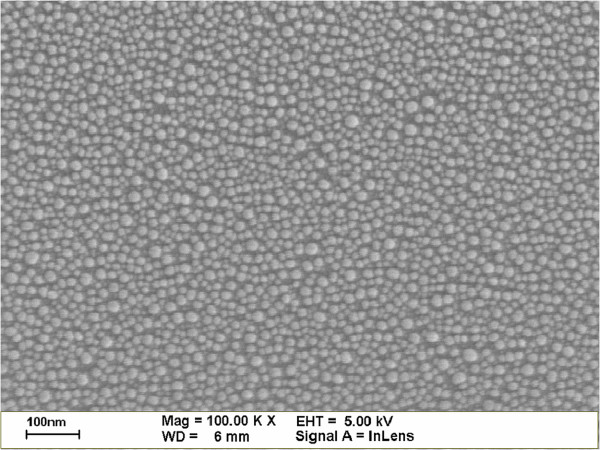
SEM image showing the sizes and profiles of In NPs.

**Figure 4:**
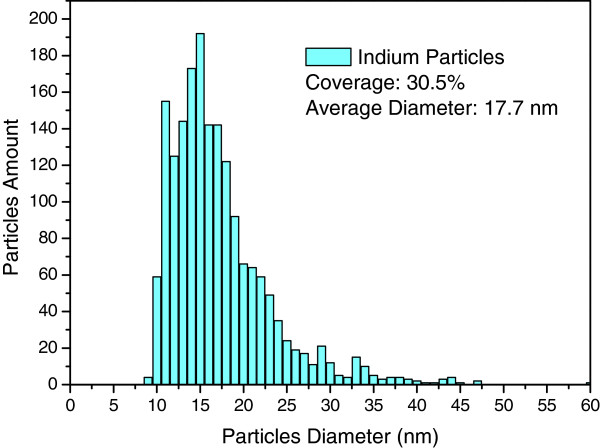
In NPs particle size distribution calculated by analyzing the SEM image using J-image software.

**Figure 5:**
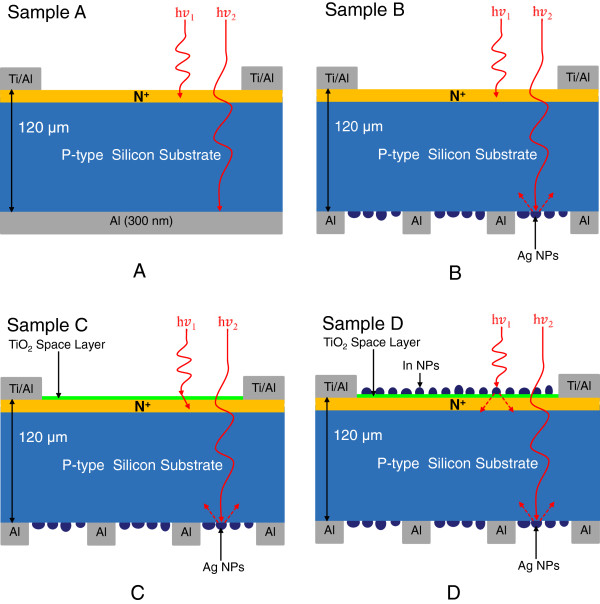
Schematic diagram of samples A (A), B (B), C (C), and D (D).

To characterize the external quantum efficiency (EQE) response of thin silicon solar cells, the samples were labeled A, B, C, and D. Sample A had a 300-nm-thick Al film deposited on the rear surface using e-beam evaporation, which was then annealed in an RTA chamber to produce a bare thin silicon solar cell. Sample B had a 300-nm-thick Al film deposited on the rear surface in grid-patterns with 60% coverage using photo-resist. This was followed by the deposition of a 30-nm-thick Ag film on the rear surface, which was annealed at 300°C under N_2_ for 3 min to form Ag NPs. The size and profile of the Ag NPs were examined using electron scanning microscopy (SEM; LEO 1530, Zeiss, Germany), the results of which are presented in Figure 1. Particle size distribution was calculated by analyzing the SEM image using J-image software, as shown in Figure 2. Sample C had a 30-nm-thick TiO_2_ spacing layer deposited on the front surface of the cell with Ag NPs and an Al electrode on the rear surface. Finally, sample D had a 3.8-nm-thick indium film deposited on the TiO_2_ layer of the cell with Ag NPs and an Al electrode on the rear surface, which was then annealed at 200°C under H_2_ for 30 min to form In NPs. This resulted in a plasmonic solar cell with In NPs on the front surface and Ag NPs on the rear surface. The size and profile of the In NPs were examined using SEM, the results of which are presented in Figure 3. Particle size distribution was calculated by analyzing the SEM image using J-image software, as shown in Figure 4. The schematic diagram of samples A, B, C, and D were given as shown in Figure 5A,B,C,D.

To examine the electrical and optical properties of the proposed solar cell, we measured the photovoltaic current-voltage (I-V) and EQE in each stage of processing. The contribution of the plasmonic scatterings of In NPs and Ag NPs was characterized according to EQE response at wavelengths between 300 and 1,000 nm (Enli Technology Co., Ltd., Kaohsiung City, Taiwan). The short-circuit current (*I*_sc_), open-circuit voltage (*V*_oc_), and conversion efficiency (*η*) were obtained using photovoltaic I-V measurements under one-sun AM 1.5 G (1,000 mW/cm^2^ at 25°C) solar simulation. The solar simulator (XES-151S, San-Ei Electric Co., Ltd., Osaka, Japan) was calibrated using a National Renewable Energy Laboratory (NREL)-certified crystalline silicon reference cell (PVM-236) prior to measurements.

## Results and discussions

EQE is the ratio of the number of photo-carriers collected by the solar cell to the number of photons of a given energy (wavelength) that strike the surface of a solar cell from outside. If all photons at a particular wavelength were absorbed and the resulting minority carriers were collected, then the quantum efficiency at that particular wavelength would be unity. The quantum efficiency of photons with energy below the band gap is zero. Generally, the quantum efficiency of a solar cell indicates the amount of current that the cell will produce when irradiated by photons of a particular energy (wavelength). Integrating the quantum efficiency of a cell over the entire solar energy spectrum would make it possible to evaluate the amount of current that the cell could produce when exposed to sunlight. Therefore, the short-circuit current density (*J*_sc_) of a photovoltaic device is found by convolving the EQE with AM 1.5 G solar energy spectrum

$$J_{\mathrm{sc}}=\int_{\lambda_{1}}^{\lambda_{n}}\mathrm{EQE}\left(\lambda \right)\cdot \lambda \cdot \frac{q}{\mathrm{hc}}\cdot E_{\mathrm{AM}1.5G}\left(\lambda \right)\mathrm{d\lambda}$$

where *q* is the elementary charge, *h* is Planck's constant, *c* is the speed of light in a vacuum, and *E*_AM1.5G_ is the spectral irradiance of AM 1.5 G in Wm^-2^ nm^-1^.

This paper focused on the enhancement of photovoltaic performance in a thin Si solar cell (120-μm-thick) through the introduction of plasmonic scattering using In NPs and Ag NPs. We measured improvements in the EQE of a thin Si solar cell resulting from the respective deposition of metallic nanoparticles on the front and rear surfaces at wavelengths of between 300 and 1,200 nm. Besides, the increased EQE leading to promote *J*_sc_ and *η* is also revealed in this study step by step due to *J*_sc_ proportional to EQE and *η* proportional to *J*_sc_ × *V*_oc_.

### Photovoltaic I-V and EQE response of plasmonic solar cell with Ag NPs on the rear surface

**Figure 6:**
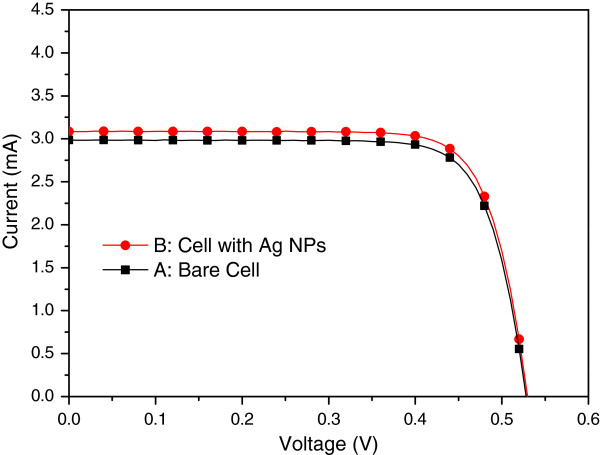
Photovoltaic I-V of bare solar cell (sample A) and cell with Ag NPs (sample B).

**Figure 7:**
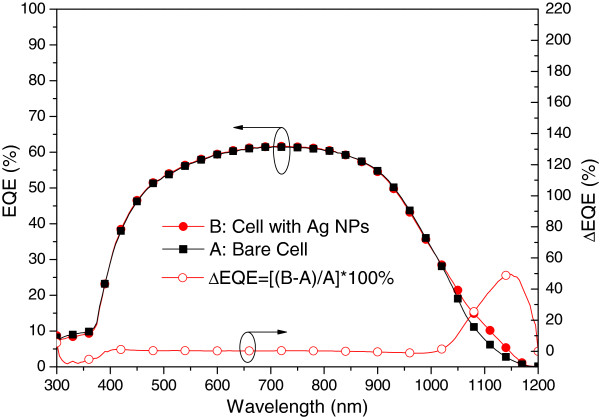
EQE of the bare solar cell (sample A) and cell with Ag NPs (sample B).

Figure 6 presents the photovoltaic I-V curves of a bare solar cell (sample A) and a plasmonic solar cell with Ag NPs on the rear surface (sample B). Under one sun AM 1.5 G illumination, the bare solar cell has an *I*_sc_ of 2.98 mA, an open-circuit voltage (*V*_oc_) of 0.53 V, and an *η* of 9.81%. In contrast, the plasmonic solar cell with Ag NPs on the rear surface presented an *I*_sc_ of 3.09 mA, a *V*_oc_ of 0.53 V, and *η* of 10.18%. The improvements in *I*_sc_ (3.69%) and *η* (3.77%) can be attributed to the plasmonic scattering produced by the Ag NPs on the rear surface. Figure 7 presents the EQE response of samples A and B. We observed a peak EQE enhancement (∆EQE) of 50% at a wavelength of 1,150 nm and ∆EQE bandwidth from 1,050 to 1,190 nm, compared with the results obtained from a bare solar cell. Thus, the increase in *I*_sc_ can be attributed to the plasmonic scattering of Ag NPs at long wavelengths, as confirmed by EQE measurements.

### Photovoltaic I-V and EQE response of plasmonic solar cell with TiO_2_ spacing layer on the front surface

**Figure 8:**
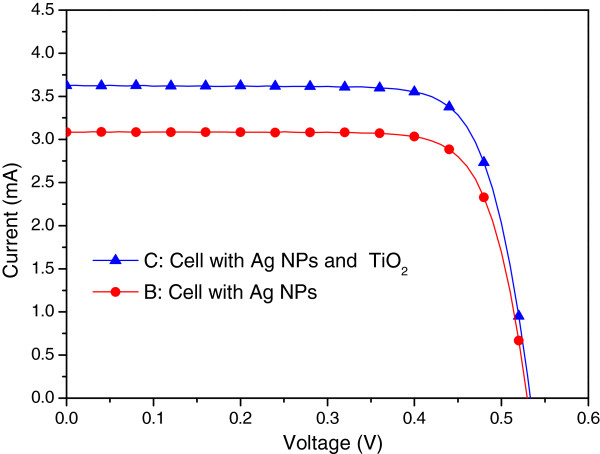
**Photovoltaic I-V curves of plasmonic solar cells of sample B and C.** Sample B: the cell with Ag NPs on the rear surface, Sample C: the cell with a 30 nm-thick TiO_2_ spacing layer on the front surface.

**Figure 9:**
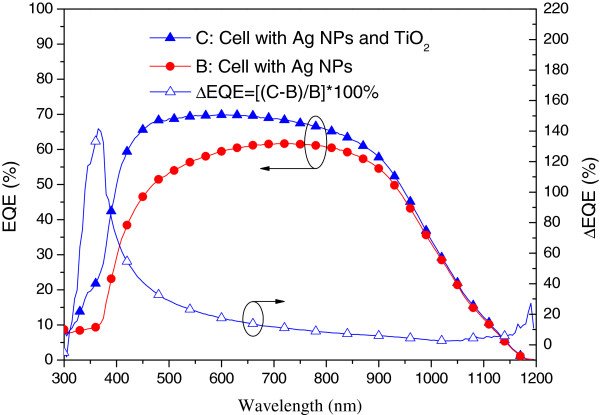
**EQE response of plasmonic solar cells of sample B and C.** Sample B: the cell with Ag NPs on the rear surface, Sample C: the cell with a 30 nm-thick TiO_2_ spacing layer on the front surface.

Figure 8 presents the photovoltaic I-V curves of a plasmonic solar cell with Ag NPs and an Al-electrode on the rear surface (sample B) as well as that of a plasmonic solar cell with a 30 nm-thick TiO_2_ spacing layer on the front surface (sample C). Under one sun AM 1.5 G illumination, sample C presented an *I*_sc_ of 3.63 mA, a *V*_oc_ of 0.53 V, and an *η* of 11.91%. The improvements in *I*_sc_ (11.50%) and *η* (16.99%) can be attributed to a reduction in the reflective loss from the surface of the solar cell due to the antireflective properties of the TiO_2_ layer. Figure 9 presents the EQE response of samples B and C. An EQE enhancement (∆EQE) of >20% was observed at wavelengths between 350 and 750 nm and high EQE values (>60%) at shorter wavelengths due to a reduction in reflective loss due to the 30-nm TiO_2_ layer on the surface of the solar cell.

### Photovoltaic I-V and EQE response of plasmonic thin silicon solar cell with In NPs/TiO_2_ layer on the front surface and Ag NPs on the rear surface

**Figure 10:**
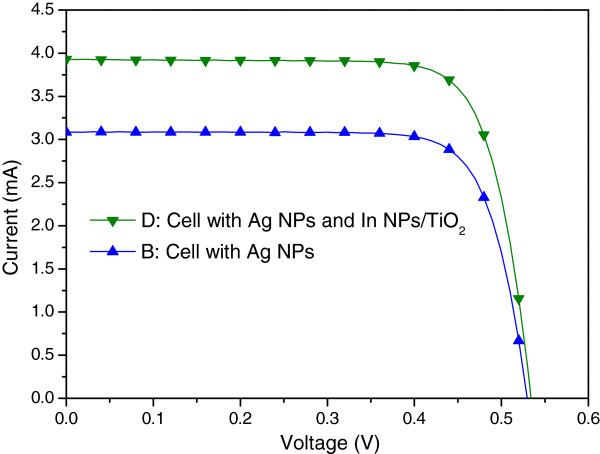
**Photovoltaic I-V curves of plasmonic solar cells of sample B and D.** Sample B: the cell with Ag NPs on the rear surface, Sample D: the cell with In NPs/TiO_2_ on the front surface as well as Ag NPs on the rear surface.

**Figure 11:**
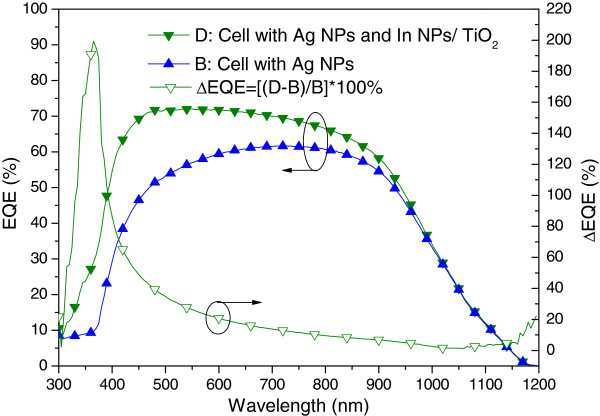
**EQE responses of plasmonic solar cells of sample B and D.** Sample B: the cell with Ag NPs on the rear surface, Sample D: the cell with In NPs/TiO_2_ on the front surface as well as Ag NPs on the rear surface.

**Figure 12:**
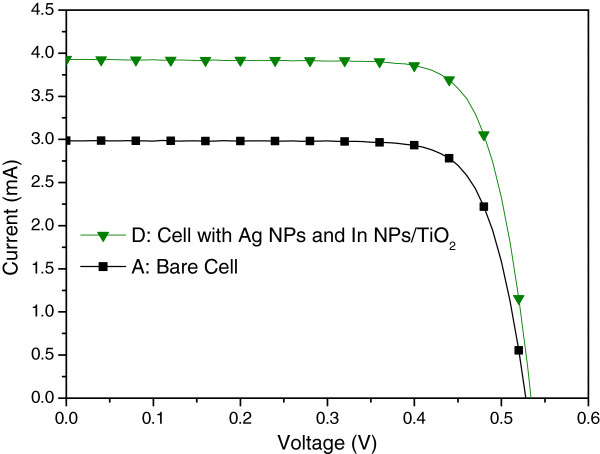
**Photovoltaic I-V curves of sample A and D.** Sample A: the bare solar cell, Sample D: the cell with In NPs on the front surface and Ag NPs on the rear surface.

**Figure 13:**
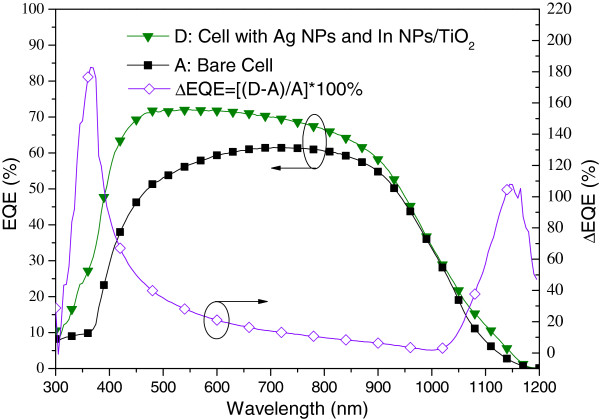
**EQE responses of sample A and D.** Sample A: the bare solar cell, Sample D: the cell with In NPs on the front surface and Ag NPs on the rear surface.

**Figure 14:**
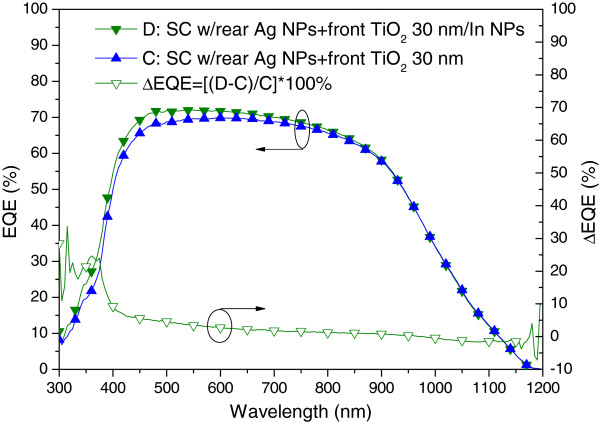
**In NPs contribution to EQE.** Which exhibited EQE enhancement of >20% between 300 and 400 nm wavelengths, compared to sample C.

Figure 10 presents the photovoltaic I-V curves of a plasmonic solar cell with Ag NPs on the rear surface (sample B) and a plasmonic solar cell with In NPs/TiO_2_ on the front surface as well as Ag NPs on the rear surface (sample D). Under one sun AM 1.5 G illumination, sample D presented an *I*_sc_ of 3.93 mA, a *V*_oc_ of 0.54 V, and an η of 13.02%. The improvements in *I*_sc_ (27.18%) and *η* (27.90%) can be attributed to the plasmonic scattering of In NPs on the front surface and Ag NPs on the rear surface. Figure 11 presents the EQE response of samples B and D. A large EQE enhancement (∆EQE >70%) was observed at wavelengths of between 350 and 400 nm, and a considerable increase in EQE compared with sample B was observed at shorter wavelengths due mainly to the scattering effects of In NPs. We can therefore attribute the increase in *I*_sc_ to the plasmonic scattering of In NPs at shorter wavelengths, which exceeded that of the Ag NPs at long wavelengths. Figure 12 and Figure 13, respectively, present the photovoltaic I-V curves and EQE response of the bare solar cell and plasmonic solar cell with In NPs on the front surface and Ag NPs on the rear surface. Overall, *I*_sc_ was enhanced by 31.59% and *η* was enhanced by 32.72%, compared to the bare solar cell. Besides, Figure 14 shows In NPs contribution to EQE which exhibited EQE enhancement of >20% between 300 and 400 nm wavelengths, compared to sample C. Table 1 summarizes the photovoltaic performance of samples A, B, C, and D.

**Table 1:** Summary of photovoltaic performance of samples A, B, C, and D

	***I***_**sc**_**(mA)**	***V***_**oc**_**(V)**	***η*** **(%)**	**∆** ***I***_**sc**_**(%)**	**∆** ***η*** **(%)**
A: Bare cell	2.98	0.53	9.81		
B: Cell with Ag NPs	3.09	0.53	10.18	3.69	3.77
C: Cell with Ag NPs and TiO_2_	3.63	0.53	11.91	17.50	16.99
D: Cell with Ag NPs and In NPs/TiO_2_	3.93	0.54	13.02	8.32	9.31
Enhancement (D-B/B) × 100				27.18	27.90
Enhancement (D-A/A) × 100				31.59	32.72

## Conclusions

This study fabricated and characterized thin silicon solar cells with different metallic nanoparticles deposited on the front and rear surfaces. The EQE response revealed plasmonic scattering at short wavelengths by the In NPs on the front surface and at long wavelengths by the Ag NPs on the rear side. Overall improvements in short-circuit current and conversion efficiency were in strong agreement with the EQE response resulting from the broadband plasmonic scattering produced by the different metallic NPs on each surface.

## Competing interests

The authors declare that they have no competing interests.

## Authors’ contributions

The work presented here was performed in collaboration of all authors. WJH figured out the mechanism about this research, participated in the analysis of data, and organized the article. YYL and SYS did the solar cells fabrication and SEM, photovoltaic I-V, and EQE measurements. All authors read and approved the final manuscript.
